# Machine learning-based integration develops biomarkers initial the crosstalk between inflammation and immune in acute myocardial infarction patients

**DOI:** 10.3389/fcvm.2022.1059543

**Published:** 2023-01-04

**Authors:** Hongyu Li, Xinti Sun, Zesheng Li, Ruiping Zhao, Meng Li, Taohong Hu

**Affiliations:** ^1^Medical College of Soochow University, The People’s Liberation Army of China (PLA) Rocket Force Characteristic Medical Center, Beijing, China; ^2^Department of Cardiovascular Medicine, Baotou Central Hospital, Institute of Cardiovascular Diseases, Translational Medicine Center, Baotou, China; ^3^Department of Thoracic Surgery, Tianjin Medical University General Hospital, Tianjin, China; ^4^Key Laboratory of Post-Neuroinjury Neuro-Repair and Regeneration in Central Nervous System, Tianjin Medical University General Hospital, Tianjin, China

**Keywords:** acute myocardial infarction, prognosis, immune infiltration, machine learning, bioinformatics

## Abstract

Great strides have been made in past years toward revealing the pathogenesis of acute myocardial infarction (AMI). However, the prognosis did not meet satisfactory expectations. Considering the importance of early diagnosis in AMI, biomarkers with high sensitivity and accuracy are urgently needed. On the other hand, the prevalence of AMI worldwide has rapidly increased over the last few years, especially after the outbreak of COVID-19. Thus, in addition to the classical risk factors for AMI, such as overwork, agitation, overeating, cold irritation, constipation, smoking, and alcohol addiction, viral infections triggers have been considered. Immune cells play pivotal roles in the innate immunosurveillance of viral infections. So, immunotherapies might serve as a potential preventive or therapeutic approach, sparking new hope for patients with AMI. An era of artificial intelligence has led to the development of numerous machine learning algorithms. In this study, we integrated multiple machine learning algorithms for the identification of novel diagnostic biomarkers for AMI. Then, the possible association between critical genes and immune cell infiltration status was characterized for improving the diagnosis and treatment of AMI patients.

## Introduction

Acute myocardial infarction (AMI) is a medical emergency caused by acute occlusion of the coronary arteries resulting in hypoperfusion and ischemic necrosis of myocardial cells. The pathophysiology of AMI is complex involving hemodynamic and circulatory dysfunction, organ failure, and even crosstalk between inflammation and immune disorders. The high incidence and mortality of AMI cause a serious social and healthy economic burden and affect the quality of human life (1, 2). It is vital to diagnose and treat AMI as soon as possible to reduce myocardial injury and malignant consequences, reduce mortality to a certain extent, and improve the patient’s prognosis (3). As of now, the evaluation of myocardial enzyme (CKMB) and cardiac troponin I (cTnI) remains the gold standard for the diagnosis of acute myocardial infarction. Nevertheless, some researchers have pointed out that patients with chronic kidney disease and heart failure also have elevated cTnI levels, making the diagnosis of AMI based on these biomarkers still unsatisfactory due to their low specificity and sensitivity (4–6). In addition, with an aging global population and an increasing life expectancy, it has become more crucial than ever to diagnose and prevent AMI.

In recent years, new technologies such as next-generation sequencing have allowed us to make great advances in diagnosing cardiovascular disease and identifying therapeutic biomarkers. With the rapid development of bioinformatics, novel methods are being developed for the prediction of AMI. It is worth noting that traditional differential gene expression analysis (DEGs) is mainly used to identify hub genes, but may lead to the loss of intrinsic biological information. Furthermore, although multi-biomarker approaches have been reported to significantly improve the diagnostic accuracy of AMI, they still lack robust capabilities due to complex genetic structures and inadequate methods (7–9). Many predictive models with poor accuracy and low efficiency may not enough for screening and early detection of AMI. Fortunately, the development of machine-learning algorithms, such as random forest (RF) and support vector machine-recursive feature elimination (SVM-REF), have been successfully applied to biomarker discovery and to build accurate prognostic risk models (10, 11).

Hence, in the present study, we integrated weighted gene co-expression network analysis (WGCNA) and DEGs analysis to identify candidate genes related to the pathogenesis of AMI. Then by combining the utilization of multiple machine-learning algorithms including the least absolute shrinkage and selection operator (LASSO), RF, and SVM-REF, we finally obtained seven optimal feature genes. Then we evaluated their predictive performance of them using the receiver operating characteristic (ROC) curve. Thereafter, the mechanism by which they contribute to AMI was investigated by functional enrichment analyses such as GO, KEGG, DO, and GSEA. Besides, immune-related algorithms such as ssGSEA were conducted to assess of the levels of infiltration of different immune cell types and functions. In conclusion, we found that seven powerful diagnostic efficacy genes were present in patients with AMI, indicating that they may provide new potential targets for diagnosis and prognosis of AMI, thus leading to improved outcomes.

## Materials and methods

### Data collection and processing

**FIGURE 1:**
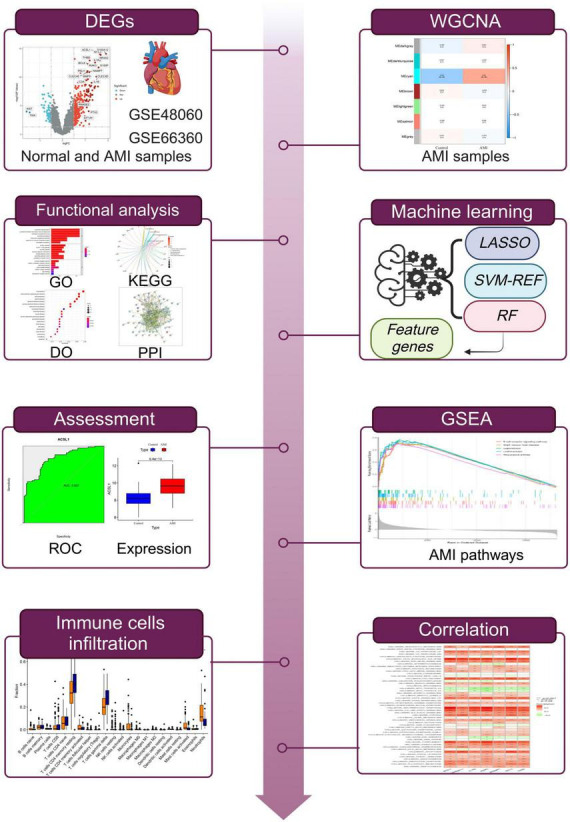
The workflow of this study.

The study flowchart is presented in Figure 1. AMI-related raw gene expression profiles data were downloaded from the Gene Expression Omnibus database (GEO).^1^ Two microarray datasets GSE48060 (GPL570, Control: 21, AMI: 31) and GSE66360 (GPL570, Control: 50, AMI: 49) were included in subsequent bioinformatics analysis. Three microarray datasets GSE19339 (GPL570, Control: 4, AMI: 4), GSE97320 (GPL570, Control: 3, AMI: 3), and GSE61145 (GPL6106, GPL6884, Control: 17, AMI: 31) were used as independent validation sets. Information on the datasets was displayed in Supplementary Table 1. It should be noted that if a gene has multiple probe loci during the conversion of probe ID and gene symbol, we use the average value of probe loci as the gene expression level. A further step was taken to convert the probe IDs to the gene symbols based on the annotation files from the respective platforms and to remove the probes which did not correspond to the gene symbols. Next, the microarray data were transformed into log2 values for further analysis. And we integrated them using Combat algorithm implemented in R package “sva” (12) and removed batch effects to form a merged dataset.

### Differential expression analysis

Differential expression analyses between AMI and control samples were conducted to identify DEGs using R package “limma” (criteria: | logFC| > 0.75, *P*-value < 0.05). Significantly upregulated genes and downregulated genes were visualized by volcano plot and heatmap.

### Weighted gene co-expression network analysis

Weighted gene co-expression network analysis was performed *via* the R package “WGCNA” to identified potential functional modules that could characterize the biological function of the AMI samples (12). It was checked to ensure that no anomalous samples had escaped clustering of samples and were excluded from the merged gene matrix. In brief, on the basis of weighted correlation adjacency matrices and cluster analyses, genes with similar expression patterns were assigned to co-expression modules. From the adjacency matrix, a topological overlap matrix (TOM) was derived, based on which genes were divided into modules according to the degree of dissimilarity between them in the TOM. The cut height, minimal module size, and soft-thresholding power were set as 0.25, 50, and 24 (scale-free *R*^2^ = 0.9), respectively. Finally, gene importance (GS) and module membership (MM) were calculated. Then spearman correlation coefficients as well as the corresponding *P*-value between control, AMI groups, and functional modules were calculated by using the Spearman method. Finally, the hub module extracted the corresponding genes were selected for in-depth analysis.

### Functional enrichment analysis and protein-protein interaction network

To identify the putative significant functional terms between the AMI and control groups, we applied the gene sets enrichment analysis (GSEA) using GSEA software with reference gene set (c2.cp.kegg.v11.0.symbols) and the significance levels for enriched gene sets were determined at *q-*value [false discovery rate (FDR)] < 0.05 and *P* < 0.05 (13). The upregulated pathways had a normalized enrichment score (NES) greater than zero, whereas the downregulated pathways had a NES less than zero. We further obtained the overlapped candidate genes between DGEs and module genes based on above mentioned analyses. Venn diagrams were created using the Venn Diagrams software^2^ to display the overlap genes. Gene ontology (GO), Kyoto Encyclopedia of Genes and Genomes (KEGG), and Disease ontology (DO) enrichment analyses were performed using “clusterProfiler” and “DOSE” R packages to explore the function and pathways of the overlapped candidate genes (14). Besides, we mapped a protein-protein Interaction (PPI) network to explore the interaction of overlapped candidate genes using the online mapping tool ‘‘STRING.^3^ “ The co-expression network was plotted by using R package “igraph” to explore the correlation intensity between score genes.

### Screening optimal feature genes

We applied a combination of machine learning algorithms (LASSO, SVM-REF, and RF) to predict disease status and identify significant prognostic variables. The LASSO regression analysis tool selects variables and regularizes them simultaneously to improve the predictive capability of statistical models (14). The SVM, a supervised machine learning method, is used for regression and classification; the FRE algorithm was used to prevent overfitting while producing interpretable results (15, 16). As a result, the SVM-RFE algorithm was used to identify the gene sets with the highest discriminatory powers that would be used to identify the most appropriate feature genes. The classification tree is the basis for the RF method, which is one of the most popular approaches to various prediction problems (17). The optimal tree number was determined by the tree number with the lowest error rate and the best stability among 1–500 trees. Following this, an RF was constructed based on the selected parameter, and the important genes were selected as the key genes for AMI diagnosis based on the decreasing accuracy method (Gini coefficient). Considering the gene importance greater than 2 is a common screening criterion in the RF algorithm, which has been used in similar studies (18), the top 10 important genes (importance > 2) were chosen as the novel gene signatures for predicting prognosis in AMI. Finally, the commonly shared genes from the intersection of a couple of machine learning algorithms were the optimal feature genes.

### The expression and diagnosis significance of optimal feature genes

The expression levels of the optimal feature genes in AMI samples and control samples were calculated using Wilcoxon rank-sum test. We further validated the predictive value of the optimal feature genes using receiver operator characteristics (ROC) curves.

### Assessment of hallmark gene sets and immune cell infiltration

The CIBERSORT algorithm is a deconvolutional arithmetic on the foundation of genetics expressions, and it can be used to assess variations in a gene group within a specimen in comparison with the variations in the rest of the genes (19). The CIBERSORT algorithm was used to identify the infiltration of 22 immune cells in normal and AMI samples, and box plots were used to illustrate the immune cell composition of patients with varying immune patterns. The Wilcoxon rank-sum test was used to evaluate the differences in immune cell proportions, and *P* < 0.05 was considered statistically significant. Additionally, the relative levels of the 50 hallmark gene sets (h.all.v7.5.1.symbols.gmt) in the merge dataset were quantified using the ssGSEA algorithm (20). Additionally, Spearman’s correlations for the 50 hallmark genes sets and the optimal feature genes were calculated.

### GSEA and correlation analysis of optimal feature genes

In addition, GSEA was utilized to determine the biological significance of optimal feature genes, utilizing the gene set of ‘‘c2.cp.kegg.v11.0.symbols’’ from the Molecular Signature Database^4^ as a reference. A gene set permutation with 1,000 times was conducted for each analysis in order to obtain a normalized enrichment score. An FDR < 0.05 was regarded as significant enrichment. Besides, correlations between optimal feature gene expression levels were calculated using Pearson correlation analysis.

### Sample collection

Six AML patients and six healthy subjects of peripheral blood was stored inside 1.5 ml RNase-free tubes at −80°C until use. All blood samples were randomly sampled from the Baotou Central Hospital from August 2021 to September 2022. Diagnosis of AMI was based on the Fourth Universal Definition of Myocardial Infarction. This study was approved by the Ethics Committee of the Baotou Central Hospital and was conducted in accordance with the Declaration of Helsinki.

### RNA extraction and quantitative reverse transcription PCR (qRT-PCR)

RNA was extracted from blood samples using Trizol reagent and then cDNA was synthesized by reverse transcription using the PrimeScript™ RT Reagent Kit (RR037, TaKaRa, China) based on the manufacturer’s protocol. GAPDH was used as the internal references, then qRT-PCR was conducted using the SYBR Green PCR Kit (RR820, TaKaRa, China) based on the manufacturer’s protocol. The expression level was quantized by 2^–ΔΔCT^ mode. All reactions were repeated in triplicate. The primers used are shown in Supplementary Table 2.

### Statistical analysis

All data processing, statistical analysis, and plotting were conducted in R software (version 4.1.1) and GraphPad Prism (version 8.0.2). Wilcoxon rank-sum test or Student’s *t*-test was utilized for analyzing the difference between the two groups. The correlation between the variables was determined using Pearson’s or Spearman’s correlation test. All statistical *P-*values were two-side, and *P* < 0.05 was regarded as statistical significance.

## Results

### Identification of DEGs between control and AMI samples

**FIGURE 2:**
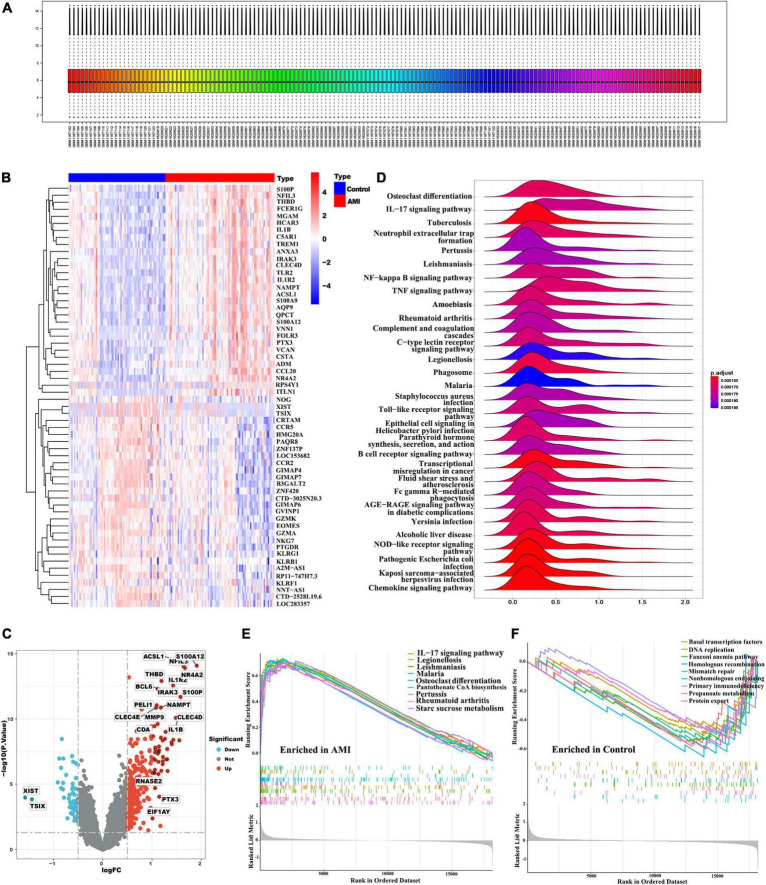
Identification of DEGs and functional annotation. **(A)** Gene expression level statistics of the integrated dataset after removed batch effect. **(B)** The heatmap of AMI-related DEGs expression levels: blue-low gene expression; red-high gene expression. **(C)** The volcano plot of AMI-related DEGs expression. **(D)** Ridgeline plot of GSEA results. **(E,F)** The main signaling pathways that are significantly enriched in the AMI group **(E)**, and in the control group **(F)**.

In this study, we merged two microarray datasets including GSE48060 and GSE66360 datasets from the GEO database and totally obtained 71 control and 80 AMI samples. Before data analysis, we removed the batch effect from different batches between the datasets (Figure 2A). Next, a total of 118 DEGs (Supplementary Table 3) including 11 downregulated genes and 107 upregulated genes were identified, which were intuitively presented in the heatmap (Figure 2B). Among them, some genes were significantly upregulated, such as ACSL1, S100A12, NFIL3, THBD, NR4A2, IL1R2, BCL6, IRAK3, S100P, PELI1, NAMPT, CLEC4E, MMP9, CLEC4D, CDA, IL1B, RNASE2, PTX3, EIF1AY, etc. While genes like XIST, TSIX were significantly downregulated (Figure 2C). To further clarify the differences in functional and biological pathways between AMI and control samples, we performed GSEA analysis of KEGG and screened significant enriched signaling pathways (Supplementary Table 4). Ridgeline plot showed that there were changes in various immune-related biological functions and processes in AMI, such as the activation of IL-17, NF-kB, and TNF signaling pathways, and the formation of centriole extracellular traps (NETs) (Figure 2D). Additionally, IL-17 signaling pathway, starch and sucrose metabolism, and pantothenate and CoA biosynthesis were significantly enriched in the AMI group (Figure 2E). In contrast, basal transcription factors, DNA replication, mismatch repair, fanconi anemia, etc. were significantly enriched in the control group (Figure 2F).

### WGCNA and screening of hub modules

**FIGURE 3:**
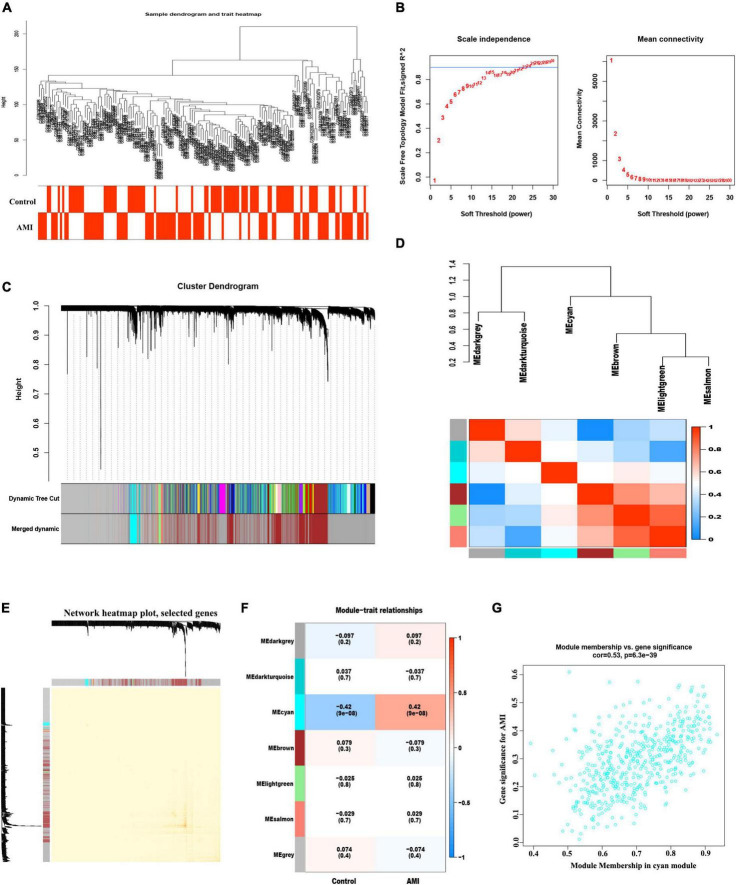
Weight correlation network analysis. **(A)** Sample clustering dendrogram with tree leaves corresponding to individual samples. **(B)** Analysis of the scale-free fit index (R^2^) and the mean connectivity for various soft-thresholding powers. **(C)** The original and combined modules under the clustering tree with cut-off values height of 0.25. **(D)** Collinear heat map of module feature genes. Red color indicates a high correlation, blue color indicates the opposite results. **(E)** Clustering dendrogram of module feature genes. **(F)** Heat map of module–trait correlations. Red represents positive correlations and blue represents negative correlations. **(G)** MM vs. GG scatter plot of AMI for cyan module.

The co-expression network was constructed by WGCNA. A total of 21,654 genes, 71 control and 80 AMI samples were preferred to cluster the samples and exclude the obviously aberrant samples by setting a threshold, as shown in Figure 3A. Then, based on scale-free *R*^2^ = 0.9 and a high average connectivity, we set the soft power threshold to 24 (Figure 3B). In total, seven modules were identified for further study after the strongly associated modules were merged according to a 0.25 clustering height limit. The primed and merged modules were eventually displayed under the clustering tree (Figure 3C). The correlation between modules was assessed, and the results revealed that there was no significant association between them (Figure 3D). The reliability of modules delineation was demonstrated by transcription correlation analysis within modules, which revealed no substantial linkage between modules (Figure 3E). Similarly, an examination of the correlation between ME values and clinical features was conducted using frontal correlations to investigated the relationships between ME values and clinical symptoms. The cyan module showed a strong correlation with AMI (*R* = 0.42, *P* < 6.3e-39) (Figures 3F, G). In total 519 candidate genes in the cyan module were included in the subsequent analysis (Supplementary Table 5).

### Functional enrichment analysis of overlapping DEGs

**FIGURE 4:**
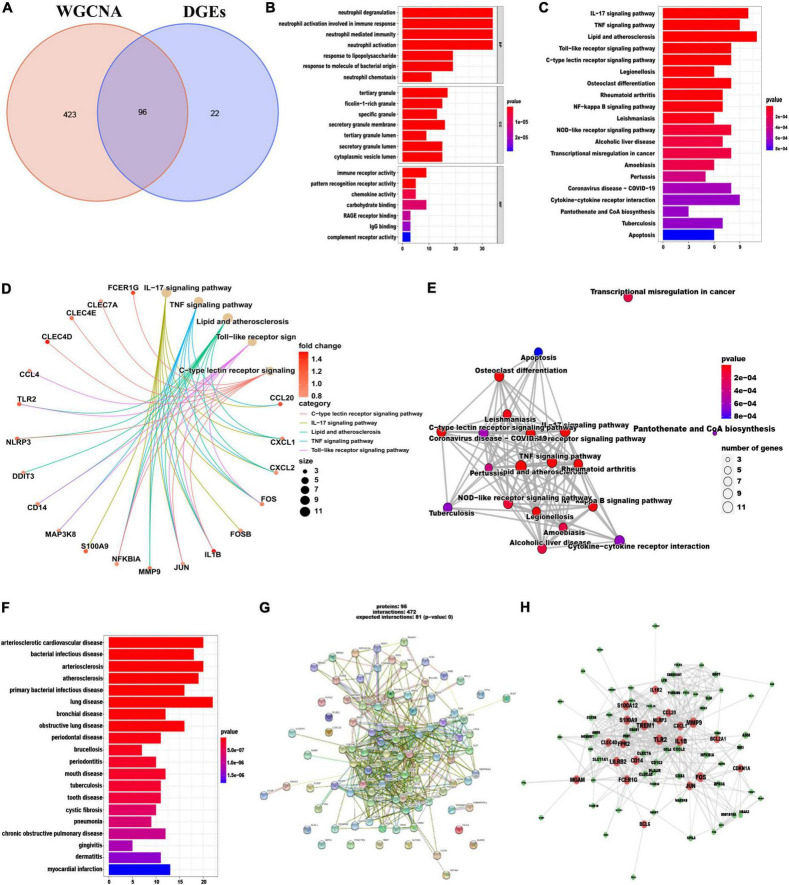
Identification and functional enrichment analyses of overlapping candidate genes. **(A)** Venn diagram showed the intersection of DEGs and module genes of WGCNA. **(B–F)** GO **(B)**, KEGG **(C–E)**, and DO **(F)** enrichment analysis of the overlapping candidate genes. **(G)** Protein-Protein Interaction (PPI) network of overlapping candidate genes. **(H)** The co-expression network showing correlation intensity of hub genes from overlapping candidate genes.

In total 96 overlapping genes (Supplementary Table 6) were screened from above mentioned DEGs and cyan module hub genes, which were also named candidate feature genes (Figure 4A). To reveal the possible biological function and enrichment pathways of the candidate feature genes, GO, KEGG, and DO analyses were carried out, subsequently. Among them, GO analysis consisted of three categories: biological process (BP), cellular component (CC), and molecular function (MF). In the BP category, the candidate feature genes were mainly enriched in neutrophil degranulation, neutrophil activation involved in immune response, neutrophil mediated immunity and neutrophil activation, etc. For the CC category, the candidate feature genes were enriched in many aspects, such as tertiary granule, ficolin-1-rich granule, specific granule and secretory granule membrane. For the MF category, the candidate feature genes were significantly enriched in immune receptor activity, pattern recognition receptor activity, chemokine activity and carbohydrate binding (Figure 4B). In addition, these genes were particularly associated with IL-17 signaling pathway, TNF signaling pathway, lipid and atherosclerosis, toll-like receptor signaling pathway, C-type lectin receptor signaling pathway, legionellosis, osteoclast differentiation, rheumatoid arthritis and NF-kappa B signaling pathway in the KEGG enrichment analysis (Figures 4C–E). DO analysis showed that the candidate feature genes mainly enriched in arteriosclerotic cardiovascular disease, bacterial infectious disease, arteriosclerosis and atherosclerosis (Figure 4F). The above functional enrichment analyses show that the immune system of AMI patients has changes in multiple dimensions, and it may have a common pathological process with the occurrence and progression of other autoimmune diseases. Then, to further reveal protein-protein interactions in the pathogenesis of AMI, we analyzed the protein-protein interaction (PPI) network of the candidate feature genes and constructed a PPI network using the String website. The PPI network for these proteins was shown in Figures 4G, H. Taken together, candidate feature genes play an important role in the pathogenesis of AMI.

### Identification of optimal feature genes by integrating multiple machine learning algorithms

**FIGURE 5:**
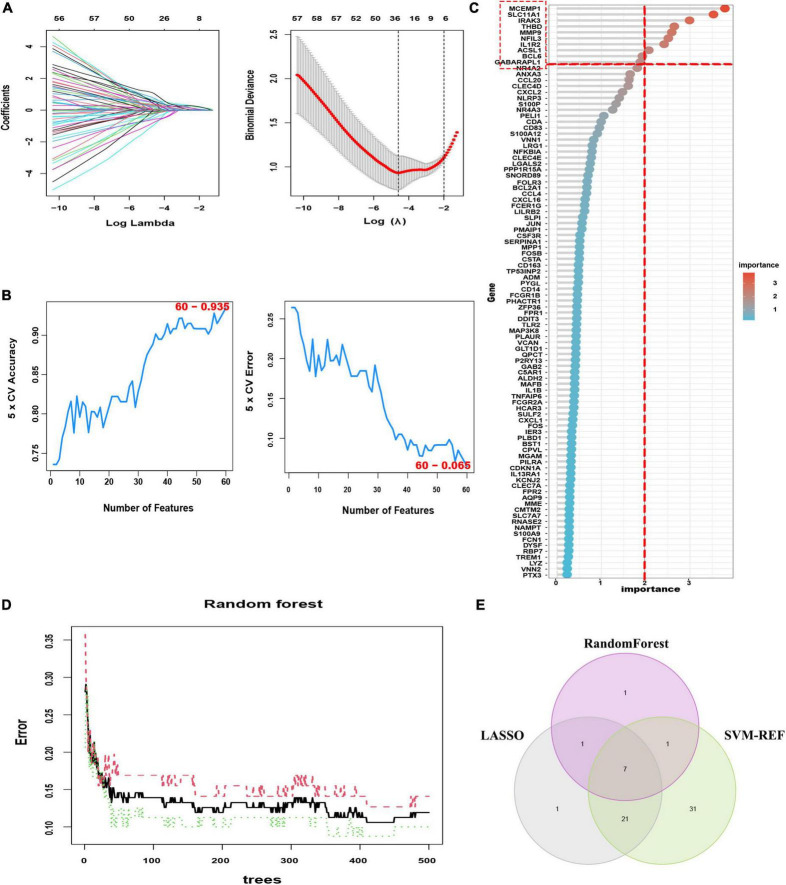
Three machine learning algorithms were integrated to identify the optimal feature genes. **(A)** LASSO coefficient profiles of the candidate optimal feature genes and the optimal lambda was determined when the partial likelihood deviance reached the minimum value. Each coefficient curve in the left picture represents a single gene. The solid vertical lines in right picture represent the partial likelihood deviance, and the number of genes (*n* = 30) corresponding to the lowest point of the cure is the most suitable for LASSO. **(B)** The SVM-RFE algorithm was used to further candidate optimal feature genes with the highest accuracy and lowest error obtained in the curves. The *x*-axis shows the number of feature selections, and the *y*-axis shows the prediction accuracy. **(C)** Relative importance of overlapping candidate genes calculated in random forest (Top 10 genes’ importance > 2). Importance indexes on the *x*-axis and genetic variables are plotted on the *y*-axis. **(D)** Random forest for the relationships between the number of trees and error rate. The *x*-axis represents the number of decision trees and the *y*-axis is the error rate. **(E)** Venn diagram showing the seven optimal feature genes shared by LASSO, Random Forest, and SVM-REF algorithms.

To identify the putative feature genes, three different machine learning algorithms were employed. Specifically, we identified 30 feature genes as the diagnostic markers for AMI form the aforementioned 96 candidate feature genes obtained from the LASSO analysis (Figure 5A and Supplementary Table 7). Furthermore, using the SVM-REF algorithm, 60 feature genes were selected after 5-fold cross-validation of the 96 candidate feature genes (Figure 5B, Supplementary Table 8). Besides, for the RF algorithm, top 10 feature genes with importance >2 were determined, including MCEMP1, SLC11A1, IRAK3, THBD, MMP9, NFIL3, IL1R2, ACSL1, BCL6, and GABARAPL1 (Figures 5C, D). Finally, the intersection of the feature genes obtained from the above three machine learning algorithms was taken and a total of seven optimal feature genes were identified, including ACSL1, GABARAPL1, IL1R2, IRAK3, MCEMP1, NFIL3, and THBD, that could be used as potential diagnostic markers for AMI and may be critical genes involved in AMI progression (Figure 5E).

### Assessment of the expression and diagnosis significance of optimal feature genes

**FIGURE 6:**
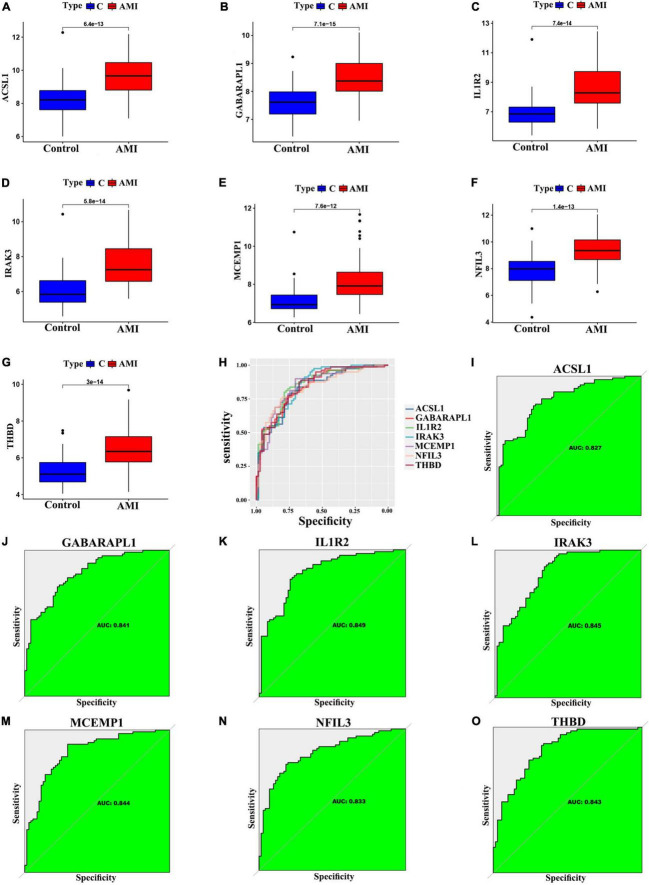
Verification of expression and diagnostic efficacy in predicting AMI progression of optimal feature genes. **(A–G)** Box plots showing the expression of ACSL1 **(A)**, GABARAPL1 **(B)**, IL1R2 **(C)**, IRAK3 **(D)**, MCEMP1 **(E)**, NFIL3 **(F)**, and THBD **(G)** in control and AMI samples. Statistic tests: Wilcoxon rank-sum test. **(H–O)** Roc curves **(H)** estimating the diagnostic performance of ACSL1 **(I)**, GABARAPL1 **(J)**, IL1R2 **(K)**, IRAK3 **(L)**, MCEMP1 **(M)**, NFIL3 **(N)**, and THBD **(O)**.

We further validated the expression levels of the 7 optimal feature genes in 80 AMI samples and 71 normal samples. Additionally, the expression levels of the 7 genes were significantly upregulated in the AMI samples, indicating their potential roles during the progression of AMI (Figures 6A–G, *P* < 0.01). Besides, to quantitatively assess the diagnostic and predictive value of the optimal feature genes, we conducted a ROC curve analysis (Figure 6H). The AUC values of ROC curves were ACSL1 of 0.827 (Figure 6I), GABARAPL1 of 0.841 (Figure 6J), IL1R2 of 0.849 (Figure 6K), IRAK3 of 0.845 (Figure 6L), MCEMP1 of 0.844 (Figure 6M), NFIL3 of 0.833 (Figure 6N), THBD of 0.843 (Figure 6O), demonstrating that these optimal feature genes enable to estimate the progression and had a high diagnostic value for AMI.

**FIGURE 7:**
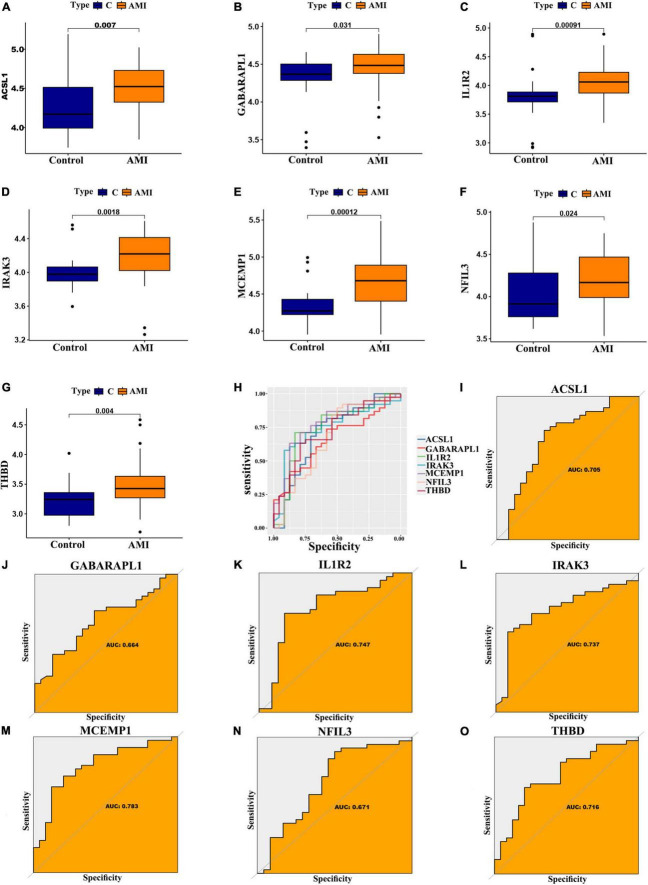
Verification of expression and diagnostic efficacy for optimal feature genes using external validation dataset. **(A–G)** Box plots showing the expression of ACSL1 **(A)**, GABARAPL1 **(B)**, IL1R2 **(C)**, IRAK3 **(D)**, MCEMP1 **(E)**, NFIL3 **(F)**, and THBD **(G)** in control and AMI samples. Statistic tests: Wilcoxon rank-sum test. **(H–O)** Roc curves **(H)** estimating the diagnostic performance of ACSL1 **(I)**, GABARAPL1 **(J)**, IL1R2 **(K)**, IRAK3 **(L)**, MCEMP1 **(M)**, NFIL3 **(N)**, and THBD **(O)**.

In addition, for accurate and reliable results, we further validated the expression levels of the optimal feature genes in external validation dataset including 38 AMI samples and 24 control samples. The GSE19339, GSE97320, and GSE61145 datasets were also normalized before analysis (Supplementary Figure 1). As shown in Figures 7A–G, the expression of the seven optimal feature genes were significantly upregulated in the AMI samples relative to the control samples (all *P* < 0.05). Meanwhile, the external validation dataset also presented high AUC values: ACSL1 (AUC: 0.705), GABARAPL1 (AUC: 0.664), IL1R2 (AUC: 0.747), IRAK3 (AUC: 0.737), MCEMP1 (AUC: 0.783), NFIL3 (AUC: 0.671), THBD (AUC: 0.716) (Figures 7H–O). The results of external validation strongly proved that all optimal feature genes are involved in AMI and have a high diagnostic value for AMI.

### Identification of the function of seven feature genes

**FIGURE 8:**
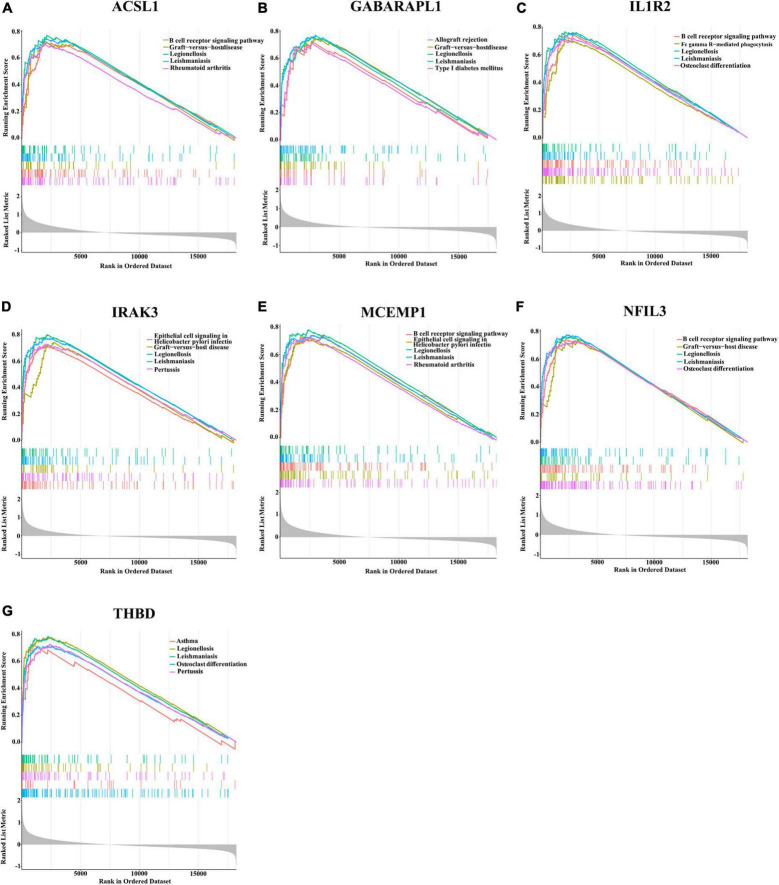
Gene sets enrichment analysis (GSEA) identifies signaling pathways in the optimal feature genes. **(A–G)** Top five signaling pathways that are significantly enriched in the high expression of ACSL1 **(A)**, GABARAPL1 **(B)**, IL1R2 **(C)**, IRAK3 **(D)**, MCEMP1 **(E)**, NFIL3 **(F)**, and THBD **(G)**.

Since these seven characteristic genes have a high guiding significance for judging prognosis, we then performed GSEA analysis on them to clarify their potential biological functions. Based on median expression levels of the optimal feature genes, we divided AMI samples into two groups, respectively. Additionally, immune-related pathways such as B cell receptor signaling pathway, graft-vs.-host disease, legionellosis, leishmaniasis, and rheumatoid arthritis were significantly enriched in the high ACSL1 subgroup (Figure 8A), while metabolism-related pathways such as butanoate metabolism, linoleic acid metabolism, and taurine and hypotaurine metabolism were significantly enriched in the low ACSL1 subgroup (Supplementary Figure 2A). Allograft rejection, graft-vs.-host disease, legionellosis, leishmaniasis and type I diabetes mellitus were significantly enriched in the high GABARAPL1 subgroup (Figure 8B), whereas metabolism of xenobiotics by cytochrome P450, aminoacyl-tRNA biosynthesis, butanoate metabolism, and valine, leucine and isoleucine degradation were significantly enriched in the low GABARAPL1 subgroup (Supplementary Figure 2B). B cell receptor signaling pathway, fc gamma R-mediated phagocytosis, legionellosis, leishmaniasis and osteoclast differentiation were significantly enriched in the high IL1R2 subgroup (Figure 8C), whereas linoleic acid metabolism, taurine and hypotaurine metabolism, and maturity onset diabetes of the young were significantly enriched in the low IL1R2 subgroup (Supplementary Figure 2C). Epithelial cell signaling in helicobacter pylori infection, graft-vs.-host disease, legionellosis, leishmaniasis and pertussis were significantly enriched in the high IRAK3 subgroup (Figure 8D), while aminoacyl-tRNA biosynthesis, primary immunodeficiency, and RNA polymerase were significantly enriched in the low IRAK3 subgroup (Supplementary Figure 2D). B cell receptor signaling pathway, epithelial cell signaling in helicobacter pylori infection, legionellosis, leishmaniasis and rheumatoid arthritis were significantly enriched in the high MCEMP1 subgroup (Figure 8E), whereas taste transduction and olfactory transduction were significantly enriched in the low MCEMP1 subgroup (Supplementary Figure 2E). B cell receptor signaling pathway, graft-vs.-host disease, legionellosis, leishmaniasis and osteoclast differentiation were significantly enriched in the high NFIL3 subgroup (Figure 8F), whereas drug metabolism - cytochrome P450, Linoleic acid metabolism, and nicotine addiction were significantly enriched in the low NFIL3 subgroup (Supplementary Figure 2F). Asthma, legionellosis, leishmaniasis, osteoclast differentiation and pertussis were significantly enriched in the high THBD subgroup (Figure 8G), whereas alanine, aspartate and glutamate metabolism, primary immunodeficiency, and ribosome were significantly enriched the low THBD subgroup (Supplementary Figure 2G). Interestingly, we noticed that B cell receptor signaling pathway was enriched multiple times, especially was enriched in the apical position in NFIL3, MCEMP1 and IL1R2 high expression groups.

### Hallmark gene sets and immune cell infiltration

**FIGURE 9:**
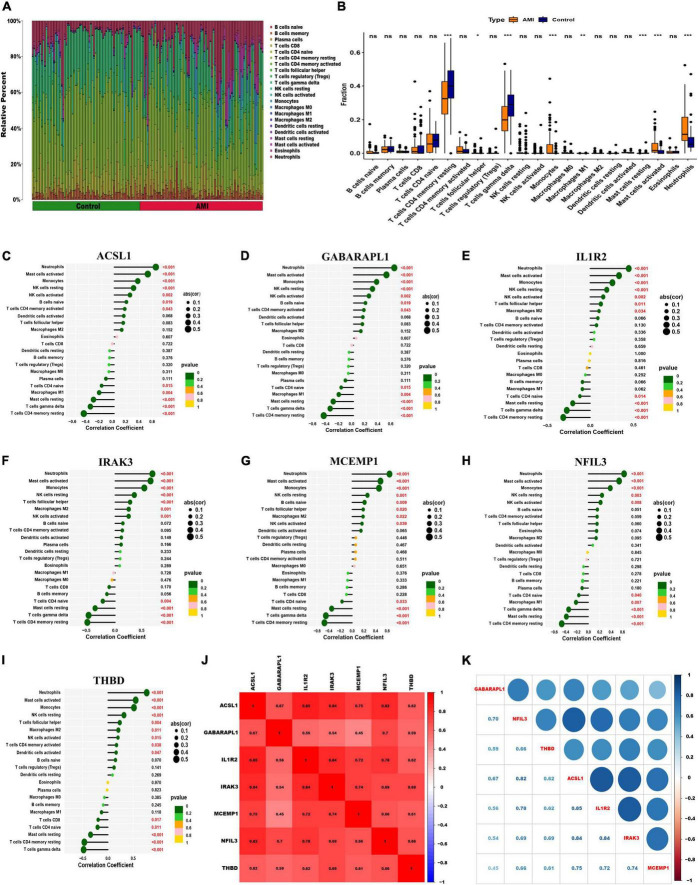
Visualization of immune cell infiltration. **(A)** The relative proportions of 22 immune cells types between control samples and AMI samples. Panel **(B)** representative boxplot shows the differences of infiltrated immune cells between control samples and AMI samples. Statistic tests: Wilcoxon rank-sum test. (*P* < 0.05*; *P* < 0.01**; *P* < 0.001***; ns, no significance). **(C–I)** Correlation between immune cells and optimal feature genes ACSL1 **(C)**, GABARAPL1 **(D)**, IL1R2 **(E)**, IRAK3 **(F)**, MCEMP1 **(G)**, NFIL3 **(H)**, and THBD **(I)**; the larger the dots, the stronger the correlation. The color of the dots represents the *P-*value; the greener the color, the lower the *P-*value. **(J,K)** Correlation analysis of seven optimal feature genes in AMI samples.

To further assess the differences in the immune cell infiltration and hallmark gene sets between AMI and control samples, the CIBERSORT algorithm was employed. The results for differential immune cell infiltration are shown in Figures 9A, B. Relative to control samples, the proportions of monocytes, mast cells activated and neutrophils were significantly upregulated in AMI samples, while the proportion of T cells CD4 memory resting and T cells gamma delta was significantly downregulated. Additionally, correlation analysis for the immune cell types with the seven optimal feature genes suggested that all seven optimal feature genes were significantly positively correlated with infiltration of neutrophils, mast cells activated, monocytes, NK cells resting, while correlated negatively with the infiltration of T cells CD4 memory resting and mast cells resting (Figures 9C–I). For example, ACSL1 gene is positively correlated with neutrophils (R = 0.65, *P* < 2.2e-16), but highly negatively correlated with T cell CD4 memory resting (R = −0.48, *P* = 6.4e-10) (Supplementary Figure 3). Gene correlations were also examined, as shown in Figures 9J, K. These seven optimal feature genes showed a significant positive correlation. For example, the correlation coefficient between ACSL1 and IL1R2 was 0.85, indicating that seven optimal feature genes had a significant functional similarity.

**FIGURE 10:**
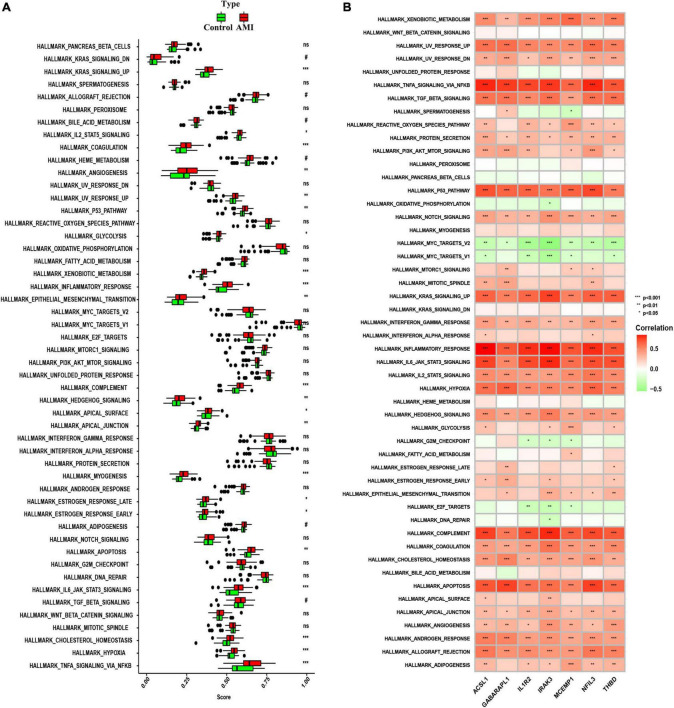
Analysis of hallmark gene sets. **(A)** The specific distribution of the 50 hallmark gene sets in AMI and control samples. **(B)** Correlation analysis of the 50 hallmark gene sets with seven optimal feature genes. Statistic tests: Wilcoxon rank-sum test (*P* < 0.2^#^; *P* < 0.05*; *P* < 0.01**; *P* < 0.001***; ns, no significance).

To further investigate whether the enrichment of hallmark gene sets differs between the AMI group and the control group, we judged the significance of the difference between the two groups for 50 hallmark gene sets based on the enrichment score by using ssGSEA algorithm. The detailed distribution of the 50 hallmark gene sets between AMI and control samples was illuminated in Figure 10A. A number of hallmark gene sets exhibited a significant difference, including KRAS-signaling-up, IL2-STAT5-signaling, angiogenesis, UV-response-up, P53-pathway, glycolysis, xenobiotic-metabolism, inflammatory-response, epithelial-mesenchymal-transition, complement, hedgehog-signaling, apical-surface, apical-junction, myogenesis, estrogen-response-late, estrogen-response-early, apoptosis, IL6-JAK-STAT3-signaling, mitotic-spindle, cholesterol-homeostasis, hypoxia, and TNFα-signaling-*via*-NFKB. So, we can infer that compared with the normal group, these hallmark gene sets were over-activated in the AMI group. Additionally, we can find that the seven optimal feature genes are generally consistent in the majority of hallmark gene sets. For instance, all of the seven optimal feature genes were positively correlated with the inflammatory-response hallmark gene set. However, across a small subset of hallmark gene sets, the seven genes were not consistently correlated. For example, GABARAPL1 was positively correlated with the G2M checkpoint, while the other six genes are negatively correlated with the G2M checkpoint (Figure 10B). These data will require us further reinforce the comprehensive interrogation of the various roles of the optimal feature genes in AMI pathogenesis.

### qRT-PCR validation of optimal feature genes

**FIGURE 11:**
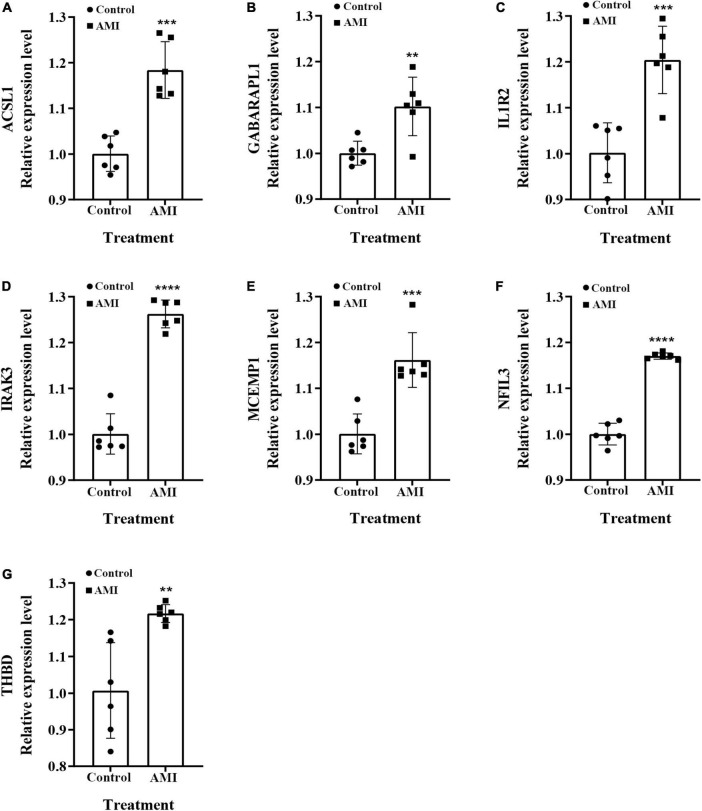
The relative expressions of optimal feature genes were validated by qRT-PCR. **(A–G)** The expressions of ACSL1 **(A)**, GABARAPL1 **(B)**, IL1R2 **(C)**, IRAK3 **(D)**, MCEMP1 **(E)**, NFIL3 **(F)**, and THBD **(G)** between AMI patients and healthy subjects. Statistic tests: Student’s *t*-test (*P* < 0.01**; *P* < 0.001***; *P* < 0.0001****).

We examined the relative expression of seven optimal feature genes in AMI patients and healthy subjects. The detailed baseline information was summarized in Supplementary Table 9. Compared to healthy subjects, the expression of ACSL1 (Figure 11A), GABARAPL1 (Figure 11B), IL1R2 (Figure 11C), IRAK3 (Figure 11D), MCEMP1 (Figure 11E), NFIL3 (Figure 11F), and THBD (Figure 11G) were significantly up-regulated in AMI patients (all *P* < 0.05), which was in line with the bioinformatics analysis.

## Discussion

The core pathological process of AMI is currently considered to be an imbalance between myocardial oxygen demand (oxygen consumption) and actual oxygen supply (21, 22). Although existing studies have pointed out that the sensitivity of the troponin-dependent AMI diagnostic method has been greatly improved, and it has shown that it can be valuable for the prognosis of AMI patients, it is a pity that this detection method is currently used for precise diagnosis and treatment (23). AMI still has some deficiencies, especially in the face of complex subtypes of AMI. Based on previous research experience, biomarkers based on the gene level are often more accurate in distinguishing the disease state of patients and can also more deeply explain the mechanism behind the disease, to guide more reasonable and effective clinical treatment strategies. Therefore, in this study, we downloaded the genetic data of AMI patients from the GEO database, used the WGCNA algorithm to find the differential genes most related to the progression of AMI disease, and comprehensively used machine algorithms such as LASSO regression, SVM-REF, and Random Forest. Finally, seven optimal feature genes (ACSL1, GABARAPL1, IL1R2, IRAK3, MCEMP1, NFIL3, and THBD) that were verified to be closely related to the diagnosis and maybe, progression, of AMI were found, and further functional enrichment analyses of these genes were carried out.

To explore the role of these seven optimal feature genes in AMI, we reviewed previous studies. Among the seven genes closely related to AMI identified in this study, three genes, ACSL1, IL1R2, and THBD, have more preliminary studies in AMI. ACSL1 (chain acyl-CoA synthase 1) encodes an enzyme that plays an important role in the activation of triglyceride synthesis (24). Early studies have shown that high expression of this gene in mouse cardiomyocytes often leads to the consequence of high myocardial triglyceride deposition, so this gene is also considered a risk factor for AMI (25). In a 2020 study, Tingting Li et al. pointed out that the same triglyceride deposition phenomenon also occurs in leukocytes overexpressing ACSL1 in the peripheral blood of AMI patients, and this process is likely to be achieved through the PPARγ pathway (26). In addition, except for participating in lipid metabolism, Yuanlong Li et al.’s study also found that the high regenerative activity of the myocardium in neonatal mice was also regulated by ACSL1 within 7 days. In the neonatal mouse MI model, mice knocked out of this gene showed more good recovery (27). IL1R2 is considered to mediate the anti-inflammatory response in the traditional inflammatory response (28). Surprisingly, the study of Amit Saxena et al. also pointed out that IL-1 can cause the infiltration of leukocytes at the AMI site and thereby prevent fibroblasts from entering the body (29). The contractile phenotype is transformed to provide a better survival microenvironment for mesenchymal stem cells, thereby improving the recovery of damaged myocardium in AMI. Similar findings were also mentioned in a clinical study by Hilde L Orrem et al. (30). It is worth mentioning that the latest study by Mingzhe Li et al. directly regarded IL1R2 as a suppressor of ischemic myocardial fibrosis and found that the main reason for the inactivation/downregulation of this gene after AMI is that its promoter region is blocked by POU2F1 (31). In addition, some research methods based on gene sequencing also pointed out that IL1R2 is closely related to AMI process (32). Interestingly, we found that the study by Enfa Zhao et al. simultaneously identified IL1R2, IRAK3, and THBD as prognostic diagnostic markers for acute myocardial infarction and found a high enrichment of the IL-17 pathway in the functional analysis, which was consistent with ours (33). The results are consistent with ours. In addition, an earlier study by Wei Chen et al. also demonstrated that another gene in the IRAK family, IRAK-M knockout mice, developed more severe ventricular remodeling and systolic dysfunction after MI (34). THBD (thrombomodulin gene) belongs to the protein C anticoagulation system, which is of great significance in maintaining the balance of hemorrhage and hemostasis in the body. Current research believes that the variation of THBD is one of the important causes of thrombosis, and coronary microthrombi Formation is also an important risk factor in the pathogenesis of AMI (35). In 2011, a clinical study by Ilaria Guella et al. pointed out that SNPs at 12 loci, including THBD, showed a high correlation with an increased risk of death after AMI (36). Unfortunately, there is still a lack of basic experimental research on the gene and the pathogenesis of AMI, but the existing clinical studies have demonstrated the potential value of this gene in AMI. The relationship between the remaining few genes and AMI has not been thoroughly studied, but some indicative studies have emerged. For example, the study of Fan Qiu et al. pointed out that GABARAPL1, by interacting with STBD1, counteracted the energy protection provided by glycoautophagy and mitophagy of OGD-treated cardiomyocytes, and aggravated myocardial injury after ischemia (37). This is consistent with the results we obtained in the ssGSEA single-gene association test. While NFIL3 (38) and MCEMP1 (39) currently with only a few omics studies demonstrated their potential relationship with AMI, our study points to the potential clinical value of both, which may be a viable direction for future research. It is worth noting that the expression levels of these key genes were verified by qRT-PCR, and the results were consistent with the results of bioinformatics.

In the analysis of immune infiltration, we found that B cells and neutrophils were deeply related to AMI. When single-gene GSEA analysis was performed, we found that the B cell receptor signaling pathway was enriched in the apical position in NFIL3, MCEMP1 and IL1R2 high expression groups. As one of the resident immune cells in the heart, during myocardial ischemia, B cells can release a variety of cytokines (including CCL2, CCL7, etc.) that chemoattract monocytes and neutrophils, thereby greatly increasing peripheral blood leukocytes myocardial infiltration (40). As early as 2013, research by Yasmine Zouggari et al. pointed out that this recruitment of B cells after MI aggravates further damage to ischemic myocardium (41). The mechanism behind this phenomenon was recently pointed out by Margarete Heinrichs et al. through the CXCL13-CXCR5 axis (42). And recently, researcher Claudia Monaco believes that B cells may be an important “middleman” in the formation of distal atherosclerosis after MI. He believes that the necrosis of cardiomyocytes can lead to the release of specific antigens that are not recognized and induce humoral immunity through B cells. Immunoglobulin deposition, which in turn leads to atherosclerosis after MI (43). A similar phenomenon was also found in the study of Tin Kyaw et al. (44). However, it is interesting that B cells are not all damaged in the biological process after MI. For example, the study by Lan Wu et al. found that after mice suffered AMI, there will be a special, mainly secreted, in the pericardial fat of mice. B cell subsets of IL-10 are infiltrated, and this group of cells exhibits anti-inflammatory and prognostic effects (45). The above studies all suggest that B cells have a strong potential in the treatment of AMI. The three genes identified in our study, which are closely related to the B cell receptor pathway, may be key to balancing the double-edged sword of B cell injury-protection. In addition, in multiple GSEA analyses, we found that the IL-17 signaling pathway was significantly enriched in AMI patients. As early as 2013, in the clinical study of Tabassome Simon et al., it was pointed out that low serum IL-17 level was the main cardiovascular time risk correlation in AMI patients (46). In the same year, the work of Onno J de Boer et al. also pointed out that IL-17A can promote thrombus formation by enhancing platelet aggregation (47). This process can feed back with the formation and release of Nets, aggravating coronary thrombosis and thus aggravating the progress of AMI (48). This is consistent with Our original GSEA analysis was consistent. Encouragingly, recent studies by Rafael Blanco-Domínguez et al. have confirmed that Th17 cells are a characteristic of AMI, and the microRNA mmu-miR-721 produced by them has diagnostic significance for AMI (48). The above evidence directly or indirectly illustrates the important role of IL-17 signaling pathway in the progression of AMI.

In addition, in the analysis of immune cell infiltration, we also found that neutrophil infiltration was significantly increased in patients with AMI. Neutrophils, as one of the most important cells in the inflammatory response, have long been considered to be involved in various stages of myocardial ischemia and coronary injury (49, 50), especially in reperfusion injury after myocardial ischemia (51). Some studies in recent years believe that neutrophils are expected to become an important target for the treatment of AMI. For example, Qing Wan et al. found that PDE4B can mediate neutrophil infiltration in mouse myocardium after AMI, and induce neutrophils to release a variety of cytokines, aggravating myocardial injury, which was obtained after administration of PDE4B inhibitors. Improve (52); Ji’e Yang et al. found that the neutrophil glycosylation product Nε- (carboxymethyl) lysine can also aggravate myocardial ischemia-reperfusion injury (53). In addition, the neutrophil extracellular traps (Nets) proposed in recent years have linked various pathological changes such as coronary thrombosis (54), coronary atherosclerosis, and myocardial inflammation in series (55). However, with further research, it has been found that neutrophils may also exhibit anti-inflammatory, pro-angiogenic and pro-reparative protective effects in AMI (56). Based on this, the regulation of neutrophils after AMI must have considerable clinical therapeutic value.

It is true that our research is based on RNA sequencing results from existing databases, and due to the data set, there will be some bias in our research results. In addition, our findings rely on bioinformatics analysis methods and simply verified the expression of these key genes by qRT-PCR, more *in vivo* and *in vitro* experiments are needed to verify the results. Taken together, our research aims to provide new ideas and directions for clinical diagnosis and precise treatment management of AMI.

## Conclusion

Overall, we found that seven powerful diagnostic efficacy genes were present in patients with AMI, indicating that they provide new potential targets for diagnosis and maybe progression of AMI, thus leading to improved outcomes. Different from other similar studies, we used more machine learning methods to enhance the accuracy of gene screening, and focused on exploring the specific genes that have the most obvious impact on AMI. It can also provide more accurate direction guidance for future AMI research. Overall, our research aims to provide new ideas and directions for clinical diagnosis and precise treatment management of AMI.

## Data availability statement

The original contributions presented in this study are included in the article/Supplementary material, further inquiries can be directed to the corresponding authors.

## Author contributions

HL, XS, ML, and TH designed the study. HL and ZL analyzed the data, participated in the data collection, and prepared the manuscript. TH and XS helped the analysis with constructive discussions. All authors critically revised the manuscript.

## Footnotes

1
http://www.ncbi.nlm.nih.gov/geo

2
http://bioinformatics.psb.ugent.be/webtools/Venn/

3
https://string-db.org/cgi/input.pl

4
http://software.broadinstitute.org/gsea/msigdb
