# Supplementary material for: Machine learning-based integration develops biomarkers initial the crosstalk between inflammation and immune in acute myocardial infarction patients
Source: Front Cardiovasc Med. 2023 Jan 4;9:1059543. doi: 10.3389/fcvm.2022.1059543 (PMC9846646; doi:10.3389/fcvm.2022.1059543)
Supplement: Supplementary file 1 [file Data_Sheet_1.docx]

Figure S1:


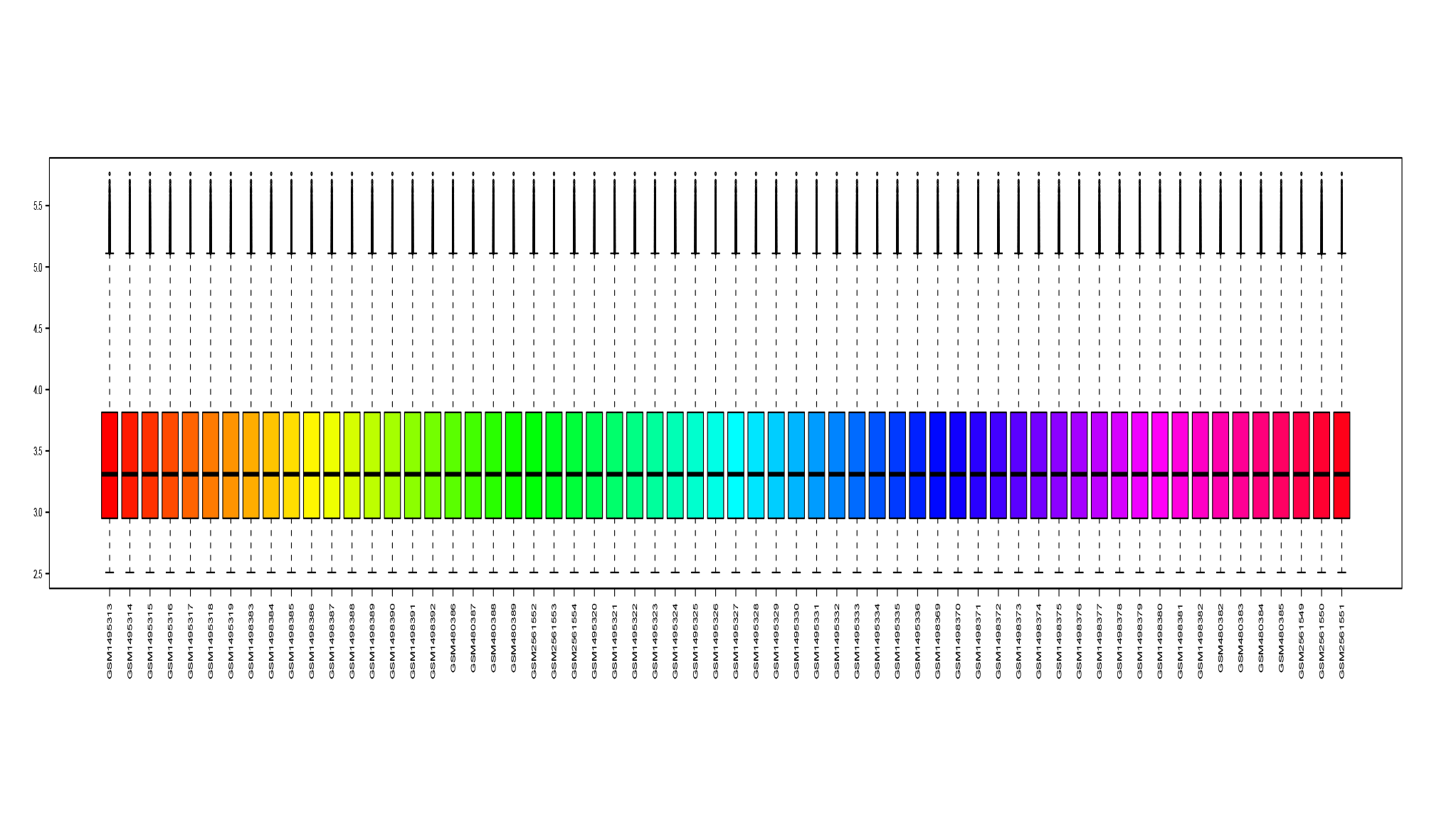


**Figure S1|** Box plot of external validation dataset after normalization.

Figure S2:


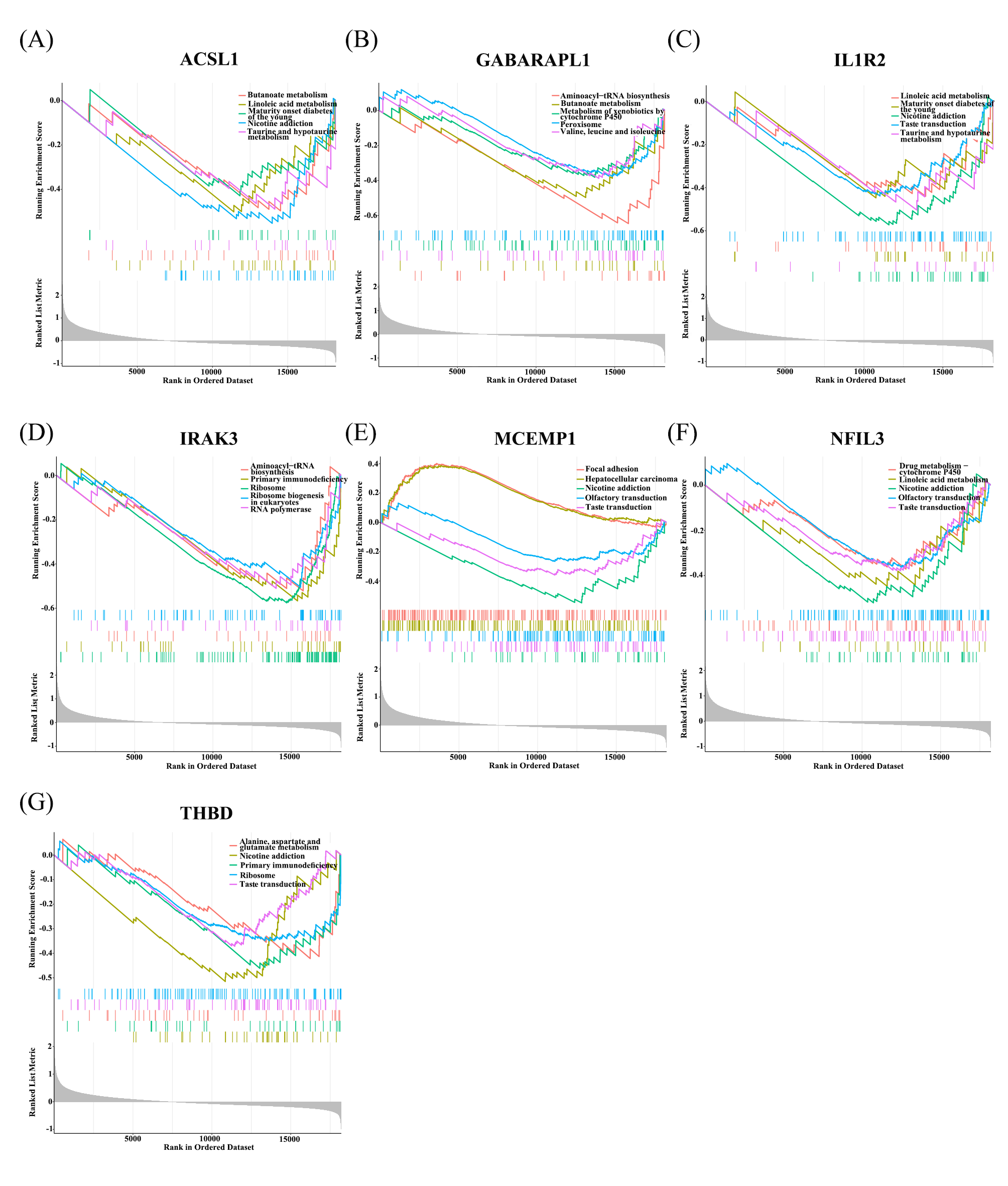


**Figure S2|** GSEA identifies signaling pathways in the optimal feature genes. (**A-G**) Top 5 signaling pathways that are significantly enriched in the low expression of ACSL1 (A), GABARAPL1 (**B**), IL1R2 (**C**), IRAK3 (**D**), MCEMP1 (**E**), NFIL3 (**F**), and THBD (**G**).

**Figure S3**


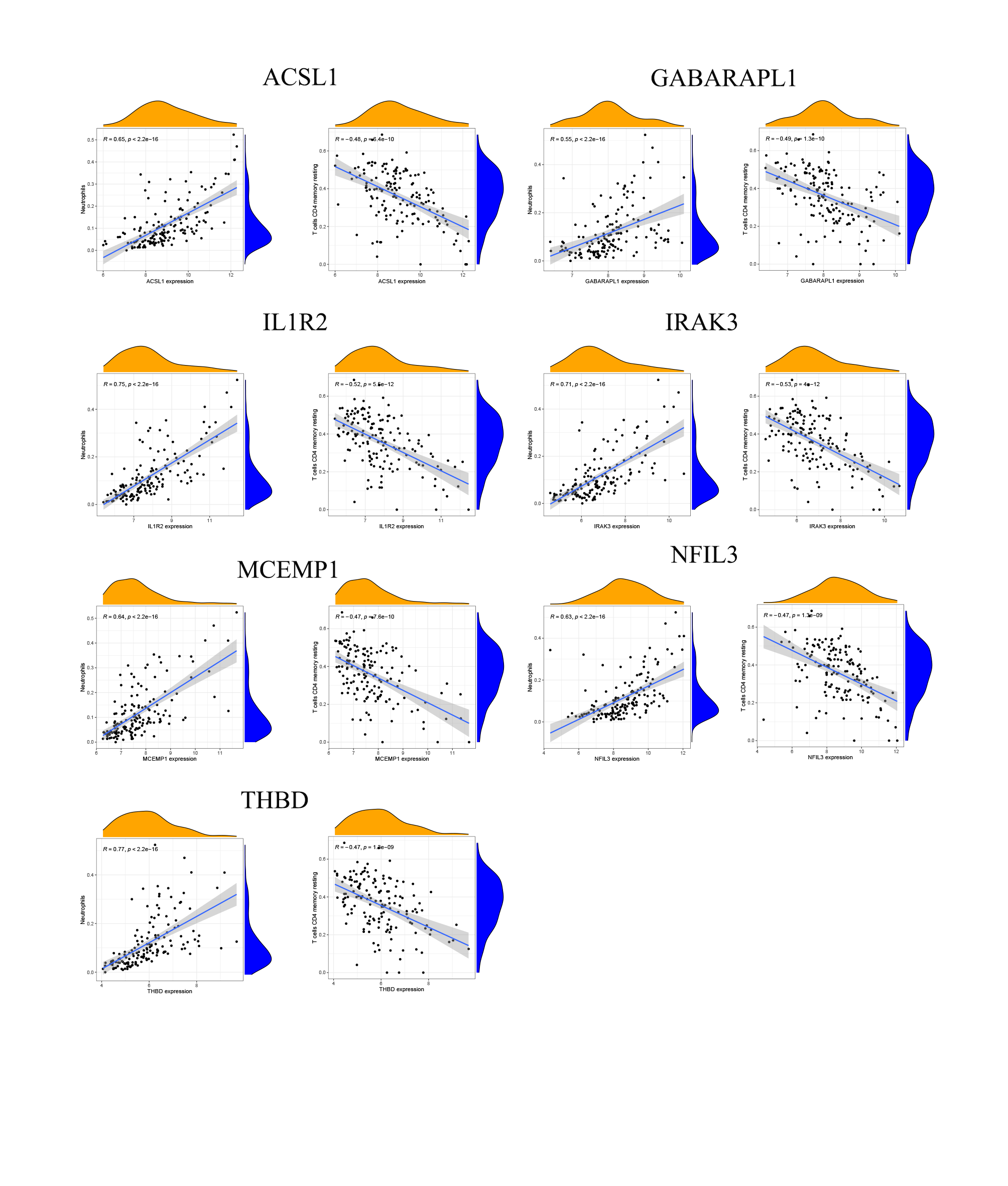


**Figure S3|**The relationship between optimal feature genes and immune cells.
